# Mismatch Between Physical and Psychological Outcomes at Return to Sport After ACL Reconstruction and the Association With Second ACL Injury Risk: A Cohort Study

**DOI:** 10.1177/19417381261442222

**Published:** 2026-05-11

**Authors:** Ramana Piussi, Jakob Lindskog, Johan Högberg, Rebecca Hamrin Senorski, Roland Thomeé, Matthew Buckthorpe, Francesco Della Villa, Kristian Samuelsson, Eric Hamrin Senorski

**Affiliations:** † Sahlgrenska Sports Medicine Center, Gothenburg, Sweden; ‡ Unit of Physiotherapy, Department of Health and Rehabilitation, Institute of Neuroscience and Physiology, Sahlgrenska Academy, University of Gothenburg, Gothenburg, Sweden; § Sportrehab Sports Medicine Clinic, Gothenburg, Sweden; ‖ Department of Orthopaedics, Institute of Clinical Sciences, Sahlgrenska Academy, University of Gothenburg, Gothenburg, Sweden; ¶ Education and Research Department, Isokinetic Medical Group, FIFA Medical Centre of Excellence, Bologna, Italy; # Faculty of Sport, Technology and Health Sciences, St Mary’s University, Twickenham, London, UK

**Keywords:** evaluation, knee, outcomes, questionnaires, reinjury, return to sport

## Abstract

**Background:**

The purpose of this study was to investigate whether a mismatch between physical function and psychological outcomes at return to preinjury level of sport (RTS) after anterior cruciate ligament (ACL) reconstruction is associated with the risk of a second ACL injury.

**Hypothesis:**

A mismatch between physical function and psychological outcomes at RTS increases second ACL injury risk

**Study Design:**

Prospective cohort (registry).

**Level of Evidence:**

Level 3.

**Methods:**

Patients registered in a local rehabilitation specific registry with ACL reconstruction, aged 15 to 40 years, who reported a preinjury Tegner level ≥6, and completed muscle function tests and patient reported outcome measures (PROs) at RTS were included. Patients were categorized into 4 our groups: (1) high physical function and high PROs (High-High), (2) low physical function and low PROs (Low-Low), (3) high physical function and low PROs (High-Low), and (4) low physical function and high PROs (Low-High). Cox regression analysis and Kaplan-Meier survival estimates were used to assess the association between group classification and second ACL injury risk within 1 year of RTS.

**Results:**

A cohort of 380 patients from was included. Within 1 year of RTS, 34 (8.9%) patients sustained a second ACL injury. The second ACL injury rate was highest in the Low-High group (19.2%). However, no statistically significant difference in hazard ratios for second ACL injury was observed.

**Conclusion:**

A “mismatch” that consists of high muscle function and low psychological status, or low muscle function and high psychological status does not appear to affect the occurrence of second ACL injury after ACL reconstruction.

**Clinical Relevance:**

Clinicians should be cautious not to rely solely on results from muscle function tests and PROs to clear patients for unrestricted sports participation.

The anterior cruciate ligament (ACL) is a primary stabilizer of the knee during cutting, pivoting, and landing movements, and second ACL injuries occur most commonly during high-load, noncontact sport-specific actions after return to preinjury level of sport (RTS). For patients who aim to RTS after an ACL reconstruction, the average incidence of a subsequent ACL injury is 15%,^38^ which raises to 26% in certain subgroups such as young active patients.^5,21^ Among patients younger than 25 years, pooled data indicate that the incidence of a second ACL injury commonly exceeds 20% after RTS, with ipsilateral graft ruptures and contralateral ACL injuries occurring at similar frequencies.^38^ Higher incidence has been reported primarily in athletes returning to high-demand pivoting sports such as football or basketball, where pooled estimates indicate second ACL injury incidences approaching 30% in younger athletes when compared with incidences <10% in cohorts with lower exposure to pivoting sports after RTS.^38^ Graft-specific incidences of second ACL injury have also been described, where rate of second ACL injuries were higher in patients with hamstring tendon autograft compared with bone-patella tendon-bone autograft.^21^

Clinicians and researchers have developed tests to facilitate RTS decision-making with the purpose of objectifying both physical and psychological state in the hope of identifying deficits that may put patients at increased risk of subsequent ACL injury after RTS.^37^ Commonly, these tests to facilitate RTS decision-making are grouped into “test batteries.” Patients who are assessed with test batteries can either pass or fail, depending on a definition, e.g., 90% symmetry in muscle function tests or a certain cut-off on a questionnaire.^27^ Questionnaires are referred to commonly as patient-reported outcome measures (PROs), and are used to capture perceived knee function and psychological aspects of recovery. These include self-reported symptoms and sport-related function, as well as constructs such as confidence or self-efficacy. Previous systematic reviews have reported inconclusively that passing test batteries to facilitate RTS decision-making can be associated with reduced rates of subsequent ACL injury,^18,37^ which may partly be due to RTS being defined inconsistently in the ACL literature, with substantial variability in how timing, level, and type of sport participation are operationalized, which limits comparability across studies and clinical applicability.^18^

However, objective measures of physical function after ACL reconstruction, such as muscle strength and hop performance, do not correlate consistently with PROs. Weak or negligible correlations have been reported between performance tests and commonly used PROs, which suggests that patients may report good knee function when objective performance remains limited, and vice versa.^1,33^ Furthermore, a scoping review highlighted methodological heterogeneity in functional testing and its association with PROs, which may contribute to the inconsistency in findings across studies.^2^ Such findings provide a conceptual basis to examine a potential mismatch between physical and psychological outcomes at RTS.

The inconclusive link between results of physical tests and PROs and the occurrence of a subsequent ACL injury after ACL reconstruction raises the question of whether a mismatch between physical function and psychological state affects the risk of a subsequent ACL injury after RTS. The definition of “mismatch” in this study was if patients had high results on physical tests and low scores on the PROs, or vice versa.

The aim of this study was to analyze the rate of subsequent ACL injury in different patient groups, with or without a mismatch between physical tests and PROs:

(1) patients who display high symmetry on physical tests and high scores on PROs,
(2) patients who display low symmetry on physical tests and low scores on PROs,
(3) patients who display high symmetry on physical tests but low scores on PROs, and
(4) patients who display low symmetry on physical tests but high scores on PROs,

at the time of RTS after ACL reconstruction.

## Methods

This study was reported according to the reporting of studies conducted using observational routinely collected data (RECORD) statement.^35^ The study was based on the Swedish rehabilitation outcome registry “Project ACL,” which aims to improve the care of patients who suffer an ACL injury independent of treatment and age.^14^ Participation is voluntary and written consent is acquired before participation in Project ACL. Patients in Project ACL are invited to regular evaluation with muscle function tests and PROs at 10 weeks, and at 4, 8, 12, 18, and 24 months, and every fifth year after ACL injury or reconstruction. Upon registration in Project ACL, patients provide demographical variables. Subsequent ACL injuries are registered by the patients or responsible physical therapists and double-checked by staff responsible for Project ACL. Project ACL has ethical approval from the Regional Ethical Review Board in Gothenburg, Sweden (registration nos. 265-13 and 265-13, T023-17), and from the Swedish Ethical Review Authority (registration no. 2020-02501).

## Test Protocol

Tests of muscle function consisted of unilateral muscle strength tests for knee extension (quadriceps) and knee flexion (hamstrings), and hop tests, which included vertical hop, hop for distance, and 30-second side hop test. Tests were supervised by trained rehabilitation staff. Patients performed a standardized warm-up: 10 minutes on a stationary bike and submaximal trials.^25^ Strength tests were performed in a seated isokinetic dynamometer at 90 deg/s (Biodex System 4), which is a reliable method (interclass correlation coefficient [ICC] = 0.95).^9^ Three maximum trials of knee extension immediately followed by knee flexion, with 40 seconds of rest between trials are performed, and maximal torque in Newton meter (Nm) is registered in Project ACL’s database. After strength tests, patients perform hop tests as the second part of muscle function assessment.^13^

During hop tests, patients are required to hop with hands held behind their back. Vertical hop was recorded as flight time from take-off to landing and converted into centimeters using the Muscle lab, Ergotest Technology. In the hop for distance, distance is measured in centimeters from toe at take-off to heel at landing. Patients had to land stably, without using arms or the opposite leg for support. For both the vertical hop and the hop for distance, 3 maximum trials are performed, with minimal rest between trials. The 30-second side hop test was performed over 2 lines 40 cm apart, where number of hops were recorded; 1 trial per leg was allowed, and 3 minutes rest between legs was included. The best result from each hop test was recorded in Project ACL’s database. Limb symmetry index (LSI) was calculated as (involved limb / uninvolved limb) × 100 for each strength and hop outcome, using the best performance from the recorded trials for each limb.

At time of test of muscle function, patients were invited to respond to PROs. Answers were collected via a web-based platform. The PROs included in Project ACL comprise the knee injury and osteoarthritis outcome score (KOOS), the knee self efficacy scale (K-SES), the ACL return to sport after injury scale (ACL-RSI), and the Tegner activity scale (Tegner).

The KOOS consists of 42 items scored on 5 subscales: pain, symptoms, activities of daily living, function in sports and recreation (Sports), and quality of life (QoL).^29^ All items are scored using a Likert scale, with 5 attainable answers from 0, extremely positive, to 4, extremely negative. Each subscale is analyzed separately with scores from 0 to 100: severe to no symptoms.^28^ The KOOS has an ICC of 0.85 to 0.9 for test-retest reliability,^7^ and there is no evidence for content validity.^10^ The psychometric properties of the KOOS have been questioned,^10^ and, when assessed with a Rasch analysis, criteria for 1-dimensionality was respected in 2 out of 5 subscales (Sports and QoL).^8^ Accordingly, only the subscales Sports, and QoL from the KOOS were used for analysis.

The 18-item version of the K-SES (K-SES_18_) aims to evaluate knee-related self-efficacy. The K-SES_18_ comprises 18 items divided into 2 subscales: present (14 items) and future (4 items) knee self-efficacy. Each item is graded from 0 to 10, with 10 being the most positive response, i.e., the greatest belief to carry out a given physical task successfully. The results from each item are added and divided by the number of items to generate a mean value for each subscale. For the short 18-item version, structural validity, internal consistency and construct validity, but not content validity, were explored.^3^ Results suggest high alpha for the future subscale (0.81-0.91), and very high alpha (0.93-0.96) for the present subscale, with risk for item redundancy, 2 factors with eigenvalues ≥1, and good construct validity.^3^

The ACL-RSI aims to measure confidence, emotion, and risk appraisal towards RTS after an ACL injury.^36^ The ACL-RSI consists of 12-items. Patients report emotion, confidence, and risk appraisal from 1 to 10, which reflects extremely negative to extremely positive response. A total score ranges between 10 and 120 and is calculated by adding response to each item and then converted to a 10 to 100 scale. The scale was found to have a Cronbach’s alpha of 0.96. Interitem correlations had a mean of 0.69 (minimum 0.49, maximum 0.83). Divergent validity analysis showed significant differences between patients who returned to sport and patients who did not.^15^ The ACL-RSI has been translated into several different languages. In the Swedish version (used in this study), Kvist et al^15^ performed a principal component analysis, which confirmed the presence of 1 underlying factor. However, when assessed with the Rasch method, the ACL-RSI showed multidimensionality and strong correlations between items.^24^

The Tegner aims to assess the level of knee-strenuous activity.^34^ The original scale ranges from 0 to 10; however, Project ACL uses a modified version which starts from level 1, where level 0 (disability due to sick leave) was removed. Higher level of Tegner indicate higher levels of knee-strenuous activity. From level 6, only sport activities are represented. Thus, if a patient rates Tegner 6 or higher it is assumed the patient is active in sport. The Tegner showed acceptable test-retest reliability (ICC = 0.8).^6^ The Tegner scores showed acceptable floor and ceiling effects and were responsive to change during rehabilitation in patients treated with ACL reconstruction.^6^

## Patients Included in the Present Study

To be eligible for inclusion, patients:

- were registered in Project ACL;
- were aged between 15 and 40 years at time of ACL reconstruction;
- were treated with primary ACL reconstruction;
- reported a preinjury Tegner ≥6;
- suffered 1 ACL injury, or 2 ACL injuries, with the latter suffered after RTS;
- participated in muscle function tests and responded to PROs at the time of RTS;
- were followed for ≥1 year after RTS.

Patients were excluded if they had not completed all muscle function tests or had not responded to all PROs at the time of RTS.

## Study Execution

The time of RTS was defined as the follow-up in which patients rated the same Tegner level as the preinjury level or higher. Data for analysis were extracted from Project ACL in October 2024. Included patients were divided into 4 groups based on the results of muscle function tests and PROs at the RTS follow-up: (1) “High-High,” (2) “Low-Low,” (3) “High-Low,” and (4) “Low-High” (Table 1). For the muscle function tests, “high” values were defined as ≥90% on the LSI on the isokinetic strength tests and the 3 hop tests, based on consensus criteria for successful outcome after ACL reconstruction.^20^ For the PROs, “high” values were defined as values equal or above recently published cut-offs calculated to differentiate patients who suffer a second ACL injury from patients who do not.^26^ For PROs, “high” values were: KOOS Sports ≥96, KOOS QoL ≥56, ACL-RSI ≥72, K-SES present ≥9.4, K-SES future ≥7 (Table 1).

**Table 1.** Groups based on results of muscle function tests and responses to PROs at time of RTS

	Muscle function	Cut-off	PROs	Cut-off
(1) High-High	High	≥90% LSI on all muscle function tests	High	On all PROs: KOOS Sports ≥96, KOOS QoL ≥56, ACL-RSI ≥72, K-SES present ≥9.4, K-SES future ≥7
(2) Low-Low	Low	<90 LSI on ≥1 muscle function test	Low	On ≥1 of the following: KOOS Sports <96, KOOS QoL <56, ACL-RSI <72, K-SES present <9.4, K-SES future <7
(3) High-Low	High	≥90% LSI on all muscle function tests	Low	On ≥1 of the following: KOOS Sports <96, KOOS QoL <56, ACL-RSI <72, K-SES present <9.4, K-SES future <7
(4) Low-High	Low	<90 LSI on ≥1 muscle function test	High	On all PROs: KOOS Sports ≥96, KOOS QoL ≥56, ACL-RSI ≥72, K-SES present ≥9.4, K-SES future ≥7

ACL-RSI, anterior cruciate ligament return to sport after injury scale; KOOS, knee injury and osteoarthritis outcome score; K-SES, knee self efficacy scale; LSI, limb symmetry index; PROs, patient-reported outcome measures; QoL, quality of life; RTS, return to preinjury level of sport.

## Outcomes

The main outcome in this study was the occurrence of a second ACL injury. Patients were stratified into groups at the time at which they performed tests and reported to RTS. Patients were then followed for 12 months after RTS to monitor the occurrence of a subsequent ACL injury. The rate of a subsequent ACL injury based on group was calculated.

Time-at-risk for the survival analyses started at the date of the follow-up at which return to preinjury Tegner level was first reported (RTS) and patients were censored 12 months after RTS if no second ACL injury occurred. A second ACL injury was defined as an ipsilateral graft rupture or a contralateral ACL injury registered in Project ACL during follow-up. The 12-month follow-up period was chosen to capture early second ACL injuries after RTS, which represents the period of highest reinjury incidence reported in registry-based cohorts.^38^

## Statistical Analysis

Statistical analyses were performed with the Statistical Analysis System, SAS Statistics for Windows, Version 9.4 (SAS Institute Inc). Means and standard deviations are presented with 95% CI for all parametric variables. Where appropriate, medians were presented with ranges from minimum to maximum, as well as first and third quartiles. Frequencies and proportions were calculated for all categorical variables. For comparison of demographic variables across groups, chi-square test was used for nonordered categorical variables, and the Kruskal-Wallis test was used for continuous variables. To calculate the hazard for a subsequent ACL injury, a Cox regression analysis was performed, with group belonging as an independent variable, and a second ACL injury as the dependent variable. The Cox analysis was adjusted for eventual confounders, that is, significant differences between groups in demographic variables. Group 1 “High-High” was used the reference group. In addition, a survival analysis for the year after return to preinjury level of sport was performed with a Kaplan-Meier estimate. The alpha level was set to < 0.05.

## Results

**Figure 1.**
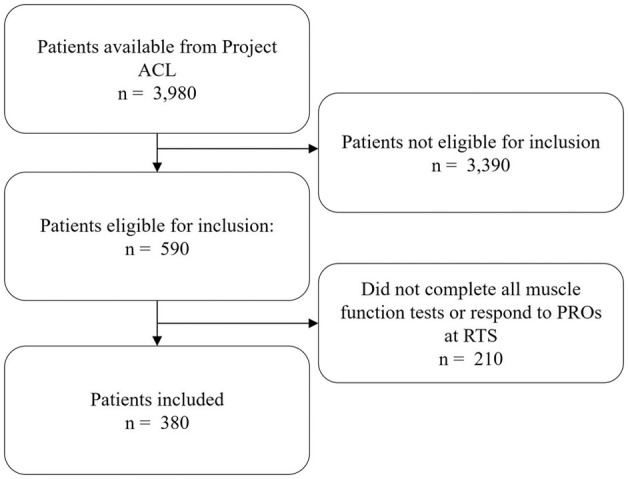
Flowchart of inclusion. ACL, anterior cruciate ligament; PROs, patient reported outcomes; RTS, return to preinjury level of sport.

A total of 380 patients were included in the present study (Figure 1), of which 48.7% were men. Included patients had an average age of 22.4 ± 6.1 years at time of surgery (Table 2).

**Table 2.** Patient demographics and comparisons between groups

	All patients	High-High	Low-Low	High-Low	Low-High	*P* value
Number	380	17	235	102	26	
Male sex, n (%)	185 (48.7%)	7 (41.2%)	107 (45.5%)	56 (54.9%)	15 (57.7%)	0.29
Age at surgery, years	22.4 (6.1)	19.6 (3.3)	22.6 (6.0)	22.1 (6.3)	23.0 (7.0)	0.27
Height, cm	175.7 (9.0)	176.5 (10.7)	175.2 (9.0)	176.6 (9.0)	175.9 (7.5)	0.50
Weight, kg	72.5 (11.7)	71.0 (10.5)	72.1 (11.8)	73.5 (11.2)	73.5 (13.4)	0.69
BMI, kg/m^2^	23.4 (2.6)	22.7 (2.1)	23.4 (2.7)	23.5 (2.4)	23.7 (3.2)	0.83
Graft choice						
Hamstring tendon	308 (81.1%)	15 (88.2%)	182 (77.4%)	88 (86.3%)	23 (88.5%)	0.24
Patellar tendon	66 (17.4%)	2 (11.8%)	48 (20.5%)	14 (13.7%)	2 (7.7%)	
Other	6 (1.5%)	0	5 (2.1%)	0	1 (3.8%)	
Preinjury Tegner						
6	25 (6.6%)	0	18 (7.7%)	7 (6.9%)	0	0.59
7	73 (19.2%)	1 (5.9%)	45 (19.1%)	22 (21.5%)	5 (19.2%)	
8	120 (31.6%)	7 (41.2%)	77 (32.8%)	30 (29.4%)	6 (23.1%)	
9	116 (30.5%)	7 (41.2%)	68 (28.9%)	32 (31.4%)	9 (34.6%)	
10	46 (12.1%)	2 (11.7%)	27 (11.5%)	11 (10.8%)	6 (23.1%)	
Time between ACL reconstruction and return to preinjury Tegner, months	15.4 (12.3)	15.1 (4.3)	14.6 (11.6)	16.3 (7.9)	19.5 (26.8)	0.0023
Subsequent ACL injury, n (%)	34 (8.9%)	2 (11.8%)	18 (7.7%)	9 (8.8%)	5 (19.2%)	0.26
Ipsilateral injury	21 (5.5%)	1 (5.9%)	10 (4.3%)	6 (5.9%)	4 (15.4%)	
Contralateral injury	13 (3.4%)	1 (5.9%)	8 (3.4%)	3 (2.9%)	1 (3.8%)	
Time between RTS and subsequent ACL injury, months	5.1 (3.3)	5.3 (0.4)	5.1 (3.4)	6.2 (3.5)	2.7 (1.9)	0.37

Data presented as mean (SD) unless otherwise stated. For comparison between groups Chi Square test was used for nonordered categorical variables and the Kruskal-Wallis test was used for continuous variables. ACL, anterior cruciate ligament; BMI, body mass index; RTS, return to preinjury level of sport.

A total of 17 (4.5%) patients were classified as High-High, 235 (61.9%) as Low-Low, 102 (26.8%) as High-Low, and 26 (6.8%) as Low-High (Table 2). Mean values for LSI and answer to PROs for the groups are presented in Appendix Table 1.

Within 1 year from RTS, 34 (8.9%) subsequent ACL injuries were recorded. The percentage of patients who suffered a second ACL injury was 11.8% in the High-High group, 7.7% in the Low-Low group, 8.8% in the High-Low group, and 19.2% in the Low-High group (Table 2).

Time from surgery to RTS differed significantly between groups; Low-High group returned later than High-High and Low-Low groups. The Cox regression analysis for the hazard rate of a subsequent ACL injury within 1 year of RTS was therefore adjusted for time between ACL reconstruction and RTS.

No significant differences in crude or adjusted hazard ratios (HRs) were observed (Table 3), despite the 19.2% injury rate in the Low-High group.

**Table 3.** Cox regression analysis of HR of a subsequent ACL injury within 1 year of RTS

		Crude		Adjusted (RTS)	
Group	Event, n (%)	HR (95% CI)	*P* value	HR (95% CI)	*P* value
High-High (Reference)	2 of 17 (11.8%)				
Low-Low	18 of 235 (7.7%)	0.64 (0.15-2.77)	0.55	0.54 (0.12-2.37)	0.41
High-Low	9 of 102 (8.8%)	0.74 (0.16-3.42)	0.70	0.74 (0.16-3.44)	0.70
Low-High	5 of 26 (19.2%)	1.79 (0.35-9.23)	0.49	1.79 (0.35-9.23)	0.49

ACL, anterior cruciate ligament; HR, hazard ratio; RTS, time to return to preinjury level of sport.

**Figure 2.**
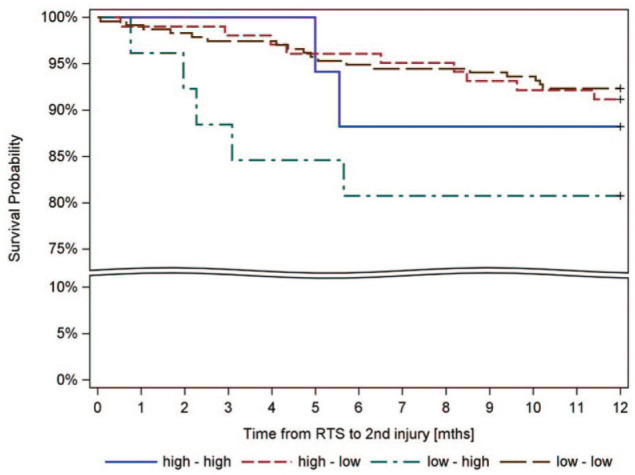
Kaplan-Meier curve with the rate of subsequent ACL injury for each of the 4 groups. ACL, anterior cruciate ligament; mths, months; RTS, return to preinjury level of sport.

Kaplan-Meier estimates showed no significant difference across groups (Figure 2).

**Figure 3.**
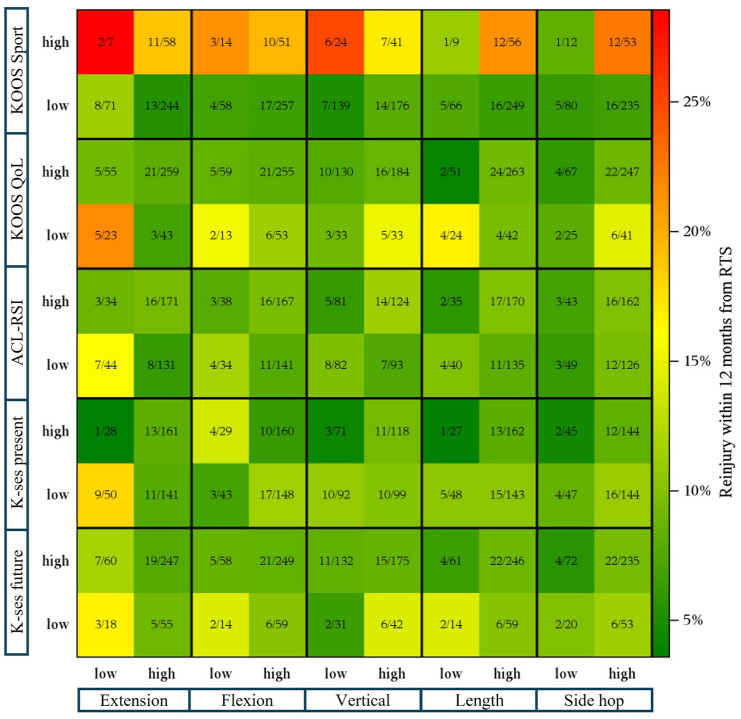
Heat map showing proportion of subsequent ACL injury (*x* axis, results on patient reported outcome measures; *y* axis, results from muscle function tests. ACL-RSI, anterior cruciate ligament return to sport after injury scale; Hams, hamstrings (isokinetic strength test); KOOS, knee injury and osteoarthritis outcome score; K-SES, knee self-efficacy scale; Length, hop for distance; QoL, quality of life; Quad, quadriceps (isokinetic strength test); Vertical, vertical hop.

**Figure 4.**
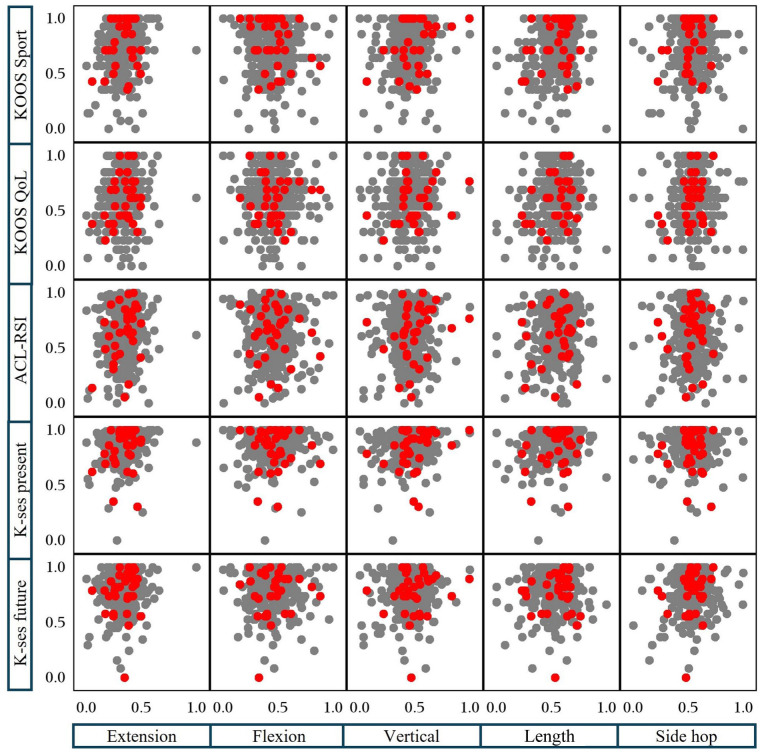
Scatterplot of subsequent ACL injury (*x* axis, PROs; *y* axis, results from muscle function tests). Red dots, subsequent ACL injury; gray dots, no subsequent ACL injury. 0.0 and 1.0 indicate the lowest and highest possible scores, respectively, on each test. ACL-RSI, anterior cruciate ligament return to sport after injury scale; Hams, hamstrings (isokinetic strength test); KOOS, knee injury and osteoarthritis outcome score; K-SES, knee self-efficacy scale; Length, hop for distance; QoL, quality of life; Quad, quadriceps (isokinetic strength test); Vertical, vertical hop.

In Figure 3 and Figure 4, the proportion of second ACL injury is plotted depending on whether patients achieved high or low PROs scores (*x* axis), and high or low muscle function (*y* axis), respectively (Figure 3). Figure 4 displays a scatter plot where all second ACL injuries are marked in red, while patients who did not sustain a second ACL injury are marked in gray. Values on *x* and *y* axes for each box are normative values where 0 represents the lowest possible score and 1 the highest possible score for respective muscle function test and PRO. The purpose of Figures 3 and 4 is to provide a descriptive visualization of how second ACL injuries are distributed across combinations of physical function and PRO scores.

For example, in Figure 3, reinjury proportions ranged from 2 of 7 (28.6%) to 1 of 12 (8.3%) among patients with high KOOS Sport scores and high muscle function, and from 8 of 71 (11.3%) to 5 of 80 (6.3%) among those with low KOOS Sport scores and low muscle function, depending on the specific test combination. In Figure 4, patients who sustained a second ACL injury were distributed across the full spectrum of normalized physical and psychological scores, without clear clustering at low or high values.

## Discussion

The main finding in the present study was that the HR for second ACL injury did not differ between the reference and the 3 predefined groups based on combinations of high or low muscle function and high or low psychological outcomes at time of RTS, which indicates that meeting or failing to achieve the cut-offs on muscle function tests and PROs, or having a mismatch between high physical function and low psychological status (or vice versa), did not affect the occurrence of second ACL injury after ACL reconstruction.

Visual inspection of Kaplan-Meier curves suggested possible increased vulnerability in the Low-High group. Although the Low-High group demonstrated the highest observed incidence of second ACL injury, the Cox regression analysis did not reveal statistically significant differences compared with the reference group. This likely reflects limited statistical power due to small and unequal group sizes, and thereby reduced precision of the hazard estimates. Consequently, the observed differences should be interpreted as descriptive and hypothesis-generating rather than confirmatory, and cannot be generalized beyond the present cohort.

Although HRs did not differ significantly, 62% of patients were classified in the Low-Low group, suggesting persistent deficits in both muscle function and PROs. Figures 3 and 4 illustrate individual test scores and second ACL injury distribution for independent interpretation.

Quadriceps strength has been reported as protective for second ACL injury after ACL reconstruction,^12,31^ but may also reflect other knee impairments.^30^ Both low and high PRO scores have been associated with reinjury,^19,39^ and fear or overconfidence may influence risk.^16^

The value of RTS test batteries remains inconclusive, likely due to variability in components, timing, and cut-offs.^17,37^ For instance, the 90% LSI threshold lacks validation as a reliable marker for a subsequent ACL injury.^32^ Evidence suggests that LSI alone may not fully capture the neuromuscular demands required to RTS with as low risk of second ACL injury as possible.^32^ The PROs used in this study, such as the ACL-RSI, the K-SES, and the KOOS, have been reported not to effectively discriminate between patients at higher or lower risk for second ACL injury.^26^ This limits clinical applicability and highlights the need for individualized decision-making.

Vertical hop had the lowest LSI, reflecting neuromuscular demands and quadriceps weakness after ACL reconstruction.^4,23^ Given the high demands on quadriceps explosiveness, and the coordination of extension in 3 joints, required for a vertical jump, this test is likely an important component of any test battery to aid in the RTS decision making process. An additional observation was that a relatively, nonsignificant larger proportion of patients in the Low-Low group had been treated with a patellar tendon graft compared with the other groups. This could partly explain the lower physical function scores in this group. However, given the nonsignificant nature of this finding, further research is needed to determine whether graft choice plays a meaningful role in both physical and psychological recovery at RTS.

## Limitations

In this study, we classified patients into groups based on results from muscle function tests and PROs. Although the cut-off values applied were based on previously established thresholds,^20,26^ they remain somewhat arbitrary and do not fully capture the underlying continuum of functional and psychological recovery. Small score differences near thresholds may lead to disproportionate group classification. The approach to dichotomize patients into “pass” or “fail” may oversimplify the complexity of recovery, but provides a structured framework that aligns with real-world clinical practice.^26^ A further limitation concerning PROS, is that the psychometric properties of the PROs used are suboptimal, which limits certainty regarding what constructs are being measured and how accurately they reflect functional and psychological recovery after ACL reconstruction. As a result, group classification and the interpretation of “mismatch” should be viewed as indicative rather than definitive, and the findings should not be interpreted as precise thresholds for clinical decision-making. In addition, group classification was based on a single assessment at the time of return to preinjury level of sport, which does not account for the dynamic nature of physical and psychological recovery after ACL reconstruction. Patients may improve or deteriorate in either domain before or after return to sport, and a single time-point assessment may therefore misrepresent a person’s longer-term recovery status. This should be considered when interpreting the observed associations with second ACL injury risk.

Concomitant injuries at time of ACL reconstruction may influence both physical performance and answer to PROs. Information on concomitant injuries is not collected in the registry. Therefore, residual confounding cannot be excluded and should be considered when interpreting the findings.

Another aspect that requires consideration is the definition of “failure” within the test batteries used in this study. Patients categorized as “low” in either muscle function or PROs needed to have failed only 1 test to be classified as such. Existing criteria to aid in RTS decision making are based on the principle that deficits in any key domain of function may reflect incomplete recovery and increased reinjury risk.^11,20^ Accordingly, an impairment in even 1 domain could indicate lingering biomechanical or neuromuscular deficits that are not captured in other measures.^22^ To adopt a strict criterion ensures an approach that prioritizes patient safety. Future studies should explore weighted or probabilistic models. Only 17 patients were included in the High-High group, of whom 2 (11.8%) sustained a second ACL injury. Consequently, subgroup analyses were limited by small and imbalanced group sizes, resulting in wide confidence intervals and limited statistical power to detect differences between groups. As such, the absence of statistically significant differences should not be interpreted as evidence of equivalence between groups. Future research with larger cohorts could provide the statistical power needed to validate these observations and eventually provide more concrete clinical implications. Finally, RTS was defined using the Tegner scale, but information on specific sport type or exposure at RTS was not available, which may have influenced second ACL injury incidence.

Although the present study is subject to several methodological constraints, these limitations do not invalidate the findings but rather define the scope within which findings should be interpreted. The study was designed to examine whether a mismatch between commonly used physical performance tests and knee-specific PROs at the time of RTS is associated with second ACL injury, using real-world registry data and clinically applied thresholds. In this context, the absence of statistically significant associations suggests that such “mismatch”, as currently operationalized, are unlikely to provide robust or generalizable risk stratification for second ACL injury. This finding remains clinically relevant, as it questions the added value of dichotomized test batteries for reinjury prediction at return to sport.

## Conclusion

A “mismatch” that consist of high muscle function but low psychological status, or low muscle function and high psychological status, appears not to affect the occurrence of second ACL injury after ACL reconstruction. Clinicians should be cautious not to solely rely on results from muscle function tests and PROs to clear patients for unrestricted sports participation.

## Clinical Recommendation

Clinicians should not rely solely on the presence or absence of a mismatch between muscle function and psychological outcomes at RTS to determine readiness for unrestricted participation after ACL reconstruction. Strength of Recommendation (SORT): B; based on limited-quality, patient-oriented evidence from a single prospective cohort study.

## Supplemental Material

sj-docx-1-sph-10.1177_19417381261442222 – Supplemental material for Mismatch Between Physical and Psychological Outcomes at Return to Sport After ACL Reconstruction and the Association With Second ACL Injury Risk: A Cohort Study Supplemental material, sj-docx-1-sph-10.1177_19417381261442222 for Mismatch Between Physical and Psychological Outcomes at Return to Sport After ACL Reconstruction and the Association With Second ACL Injury Risk: A Cohort Study by Ramana Piussi, Jakob Lindskog, Johan Högberg, Rebecca Hamrin Senorski, Roland Thomeé, Matthew Buckthorpe, Francesco Della Villa, Kristian Samuelsson and Eric Hamrin Senorski in Sports Health
