# Supplementary material for: Mismatch Between Physical and Psychological Outcomes at Return to Sport After ACL Reconstruction and the Association With Second ACL Injury Risk: A Cohort Study
Source: Sports Health. 2026 May 11:19417381261442222. Online ahead of print. doi: 10.1177/19417381261442222 (PMC13167922; doi:10.1177/19417381261442222)
Supplement: sj-docx-1-sph-10.1177_19417381261442222 – Supplemental material for Mismatch Between Physical and Psychological Outcomes at Return to Sport After ACL Reconstruction and the Association With Second ACL Injury Risk: A Cohort Study [file sj-docx-1-sph-10.1177_19417381261442222.docx]

Appendix

| **Appendix Table 1: Results from muscle function tests and PROs across groups** | | | | |
| --- | --- | --- | --- | --- |
| **Table 2** | **High - High (n=17)** | **Low - Low (n=235)** | **High - Low (n=102)** | **Low - High (n=26)** |
| **Muscle tests:** |  |  |  |  |
| **Quadriceps (LSI)** | 106.9 (9.2) 105.2 (100.8; 110.7) n=17 | 94.0 (10.1) 94.5 (88.6; 100.4) n=235 | 102.3 (8.5) 101.2 (98.3; 105.7) n=102 | 97.7 (9.0) 97.7 (92.1; 103.6) n=26 |
| **Hamstring (LSI)** | 101.3 (7.2) 98.8 (96.1; 107.1) n=17 | 98.3 (11.6) 97.8 (89.6; 104.7) n=235 | 102.9 (8.6) 102.2 (97.4; 107.1) n=102 | 93.3 (13.7) 93.8 (86.2; 101.8) n=26 |
| **Vertical hop (LSI)** | 103.8 (5.7) 104.1 (101.2; 107.3) n=17 | 87.3 (12.6) 87.7 (81.2; 95) n=235 | 102.3 (10.1) 100.3 (95.4; 106.3) n=102 | 87.3 (20.2) 86.2 (78.2; 96) n=26 |
| **Hop for distance (LSI)** | 101.9 (4.8) 100 (99.2; 106.3) n=17 | 93.7 (9.0) 94.1 (89.3; 99.3) n=235 | 99.4 (5.4) 99.3 (95.1; 102.4) n=102 | 92.6 (6.3) 92.4 (88.9; 97.6) n=26 |
| **Side Hop (LSI)** | 105.2 (6.3) 105.5 (101.8; 109.8) n=17 | 95.0 (16.8) 95.3 (87.5; 103.4) n=235 | 104.9 (10.8) 102 (97.8; 107.7) n=102 | 92.8 (11.5) 96.3 (87.5; 100) n=26 |
| **PROs:** |  |  |  |  |
| **KOOS Sports** | 99.8 (1.0) 100 (100; 100) n=17 | 78.4 (15.2) 80 (70; 90) n=235 | 84.3 (11.9) 87 (79; 95) n=102 | 99.7 (1.1) 100 (100; 100) n=26 |
| **KOOS QoL** | 83.6 (14.0) 88 (75; 94) n=17 | 65.1 (15.9) 69 (56; 75) n=235 | 69.6 (16.5) 69 (56; 81) n=102 | 87.8 (10.5) 88 (81; 100) n=26 |
| **ACL-RSI (o-1oo)** | 87.5 (8.2) 85.8 (81.7; 94.2) n=17 | 69.2 (17.8) 70 (57.5; 83.3) n=235 | 72.3 (16.8) 73.8 (64.2; 85.8) n=102 | 91.6 (7.6) 92.1 (87.5; 98.3) n=26 |
| **K-SES present** | 9.8 (0.1) 9.9 (9.9; 10) n=17 | 8.8 (1.1) 9.1 (8.3; 9.6) n=235 | 9.2 (0.9) 9.4 (9; 9.8) n=102 | 9.8 (0.2) 9.9 (9.7; 10) n=26 |
| **K-SES future** | 9 (0.9) 9 (8.3; 9.8) n=17 | 7.8 (1.6) 8 (7; 9) n=235 | 7.9 (1.42) 8.3 (7.3; 9.3) n=102 | 9.4 (0.7) 9.5 (9; 10) n=26 |
| For continuous variables Mean (SD) / Median (Q1; Q3) / n= is presented.  LSI = limb symmetry index;  PROs = patient reported outcome measures;  KOOS = knee injury and osteoarthritis outcome scores  ACL-RSI = anterior cruciate ligament return to sport after injury scale;  K-SES = knee self efficacy scale | | | | |
